# “It was clear from his childhood that he was destined to be a top striker”: investigating the construction of football talent narratives in the Italian sporting press

**DOI:** 10.3389/fpsyg.2026.1817389

**Published:** 2026-04-24

**Authors:** Gabriele Morganti, Thomas Blackwood, Alexandra Lascu, Francesca Strassoldo di Villanova, Elvira Padua, Bruno Ruscello, Adam L. Kelly

**Affiliations:** 1 Department of Human Sciences and Promotion of the Quality of Life, San Raffaele Roma Open University, Rome, Italy; 2 Research for Athlete and Youth Sport Development (RAYSD) Lab, Centre for Life and Sport Sciences (CLaSS), Faculty of Health, Education and Life Sciences, Birmingham City University, Birmingham, United Kingdom; 3 Institute for International Strategy, Tokyo International University, Tokyo, Japan; 4 Faculty of Health, Research Institute of Sport and Exercise, University of Canberra, Canberra, ACT, Australia; 5 Institut des Sciences du Sport-Santé de Paris, Université Paris Cité, Paris, France; 6 Department of Industrial Engineering, Faculty of Engineering, “Tor Vergata” University, Rome, Italy; 7 LUISS SportLab, LUISS University, Rome, Italy

**Keywords:** developmental advantages, ecological psychology, sociocultural constraints, talent development, talent identification, talent selection, youth football

## Abstract

**Introduction:**

In this study, we adopted an interpretivist perspective to explore the construction of football talent narratives in the Italian sporting press. We aimed to understand distal influences on the recognition of beliefs and practices related to talent identification, selection, and development in Italian football.

**Methods:**

To achieve this, we conducted inductive content analysis on 26 articles from La Gazzetta dello Sport and interpreted the findings through the Skilled Intentionality Framework.

**Results:**

Findings revealed that football talent narratives were constructed to emphasize: (a) the early steps (i.e., early start and father as mentor and gateway to early participation, early selection and specialization), (b) developmental pathways and career trajectories (i.e., early achievements and performance progression, playing up, and early exposure to senior-level football), (c) the attributes (i.e., body proportions and physical attributes, technical and tactical skills, and psychological characteristics), and (d) socially mediated validation (i.e., public recognition and market value and youth international selection).

**Discussion:**

We argue that the Italian sporting press uses its narratives not only to describe talent but also to prescribe particular ways of becoming talented, thereby guiding the intentionality of players, parents, practitioners, and other stakeholders navigating the Italian football ecosystem. In line with this, our study contributes to the ongoing discussions about refining conceptualizations of talent identification and development as complex and socially constructed processes, encouraging sporting systems to reflect on the wider sociocultural context in which they operate, to investigate and refine their current practices and outcomes from an ecological perspective.

## 1 Introduction

Despite being commonly used in everyday language by fans and media, as well as regularly employed by practitioners and researchers (Baker et al., 2020), the concept of “talent” within sport remains neither well-defined nor well-understood (Baker et al., 2024; Johnston et al., 2018; Till and Baker, 2020) and highly context-specific (Grainger et al., 2025). Research from (Jones et al. 2020) investigated how Canadian collegiate-level coaches constructed talent in their respective sports, revealing that their individual beliefs, experiences, and values, as well as the context of their sport, strongly influenced their views on talent. Johnston and Baker (2022) also researched the subjective beliefs about talent of Canadian distance running coaches, revealing that coaches expressed different preferred physical and psychological profiles for their athletes. Similarly, Koopmann et al. (2025) reported that German basketball coaches assess players by relying on instinct or experiential knowledge, which was shaped by their personal experiences, professional development, and preferences (Johnston et al., 2025). Additionally, Lund and Söderström (2017) highlighted that talent identification in Swedish football is an implicit process, whereby they used Bourdieu's (1984) concept of habitus (taste) to explain coaches' decision-making, proposing that coaches develop an embodied perception of what it entails to be a higher-level football player through their history, social experiences, and interactions within specific groups, which guides their evaluations of players. In line with this, Champ et al. (2020) suggested that football coaches can develop hegemonic beliefs, including preferences and notions about talent, which can be difficult to challenge and result from the broader institutional and cultural context.

For this reason, talent may be considered a relativist concept, constructed by individuals in relation to their lived experiences and social interactions (Jones et al., 2020). Using theoretical discourse analysis, Monsees (2025) revealed that talent in football cannot be understood as a fixed or universally shared concept, but rather an empty signifier, which gains meaning only when linked to other signifiers (e.g., environment, performance, or personality). After conducting semi-structured interviews with German and Swedish youth football practitioners, the author highlighted that notions of talent are constructed in relation to the presence (i.e., what is talent) or absence (i.e., what is not talent) of specific attributes. These attributes were articulated differently across countries, indicating that organizational constraints (Lund and Söderström, 2017; Roberts et al., 2025) or specific cultures (Christensen, 2018; Ejekwumadu, 2026; Laffer et al., 2025) can shape different coaches' preferences and expectations of athletes. Consequently, given that views about talent can significantly influence the identification, selection, and development of athletes, there is a need for greater conceptual clarity across contexts (Johnston et al., 2018; Laffer et al., 2025; O'Sullivan et al., 2025). This would allow a better understanding of how sociocultural, historical, and organizational factors interact to shape perceptions of talent, defining the inclusion/exclusion of youth players (Morganti et al., 2025b), as well as their developmental experiences and skill-acquisition processes.

Taken together, this body of knowledge underlines that constructions of talent do not emerge and develop in isolation but rather are embedded and negotiated through discourses promoted by the environment (Monsees, 2025). Consequently, researchers have started to investigate the actors who contribute to the cultural construction of its meanings at the broader societal level, including the media, which can act as a macro-environmental factor influencing talent development (Henriksen and Stambulova, 2017). As an example, newspapers can facilitate the dissemination of macrosystemic values, including regular ways of doing and being, which can directly influence collective understanding and ideals, such as a common perception of what talent is in sporting contexts and what needs to develop (Blackwood, 2010; Hobsbawm and Ranger, 1983; Morganti et al., 2025b; O'Sullivan et al., 2023). For instance, Monsees (2024) found differences in how German and Swedish newspapers construct discourses around football talent. These discrepancies may reflect broader cultural differences that shape how players are perceived across contexts, suggesting that the sporting press can play a role in shaping and reinforcing those preferences.

Accordingly, in this study, we aimed to explore how the Italian sporting press constructs their football talent narratives to understand the sociocultural and historical influences that contribute to the production and reproduction of specific beliefs, habits, and practices related to talent identification, selection, and development processes in Italian football.

We structured the remainder of the paper as follows. First, we outline our methodological approach, including our philosophical orientation, the theoretical framework, and the data sources, data collection, and analytical procedures. We then report the findings that emerged from our analysis, which are interpreted and discussed in relation to the existing literature in an integrated section to maintain coherence in the narratives. Finally, we provide a concluding section that offers an overall understanding of the football talent narratives in the Italian sporting press and highlights broader implications for talent identification and development.

## 2 Methodological approach

### 2.1 Philosophical orientation

This study is guided by the philosophical assumptions of interpretivism, which does not conceive the world as constituted by fixed objects but rather asserts that reality and our understanding of it are social products, and therefore cannot be comprehended independently of the social actors, including researchers, who create it and interpret its meaning (Berger and Luckmann, 1966; Orlikowski and Baroudi, 1991; Smith, 1983). For this reason, interpretive inquiry aims to uncover sociocultural derived, contextually and historically situated understandings of the social world (Klein and Myers, 1999; Madill et al., 2000; Potrac et al., 2014).

Consistent with our philosophical orientation, we clarify the ontological and epistemological foundations for our study. Ontologically, we adopted the position of constructionism, meaning that (social) reality is socially constructed (Orlikowski and Baroudi, 1991; Sparkes, 1992). This position recognizes how individuals belonging to social groups participate and interact in social processes, characterized by meanings, beliefs, and intentions, as well as influenced by a series of historical, political, and cultural factors, which constitute the production and reproduction of specific actions, practices, and experiences (Dewar, 1991; Potrac et al., 2014). Epistemologically, we assumed the position of subjectivism, whereby knowledge was constructed and sustained involving interactions between us, the researchers, and the researched, rather than being objectively discovered or neutrally observed (Coe, 2021; Sparkes, 1992). This way, the aim was not to establish a universal truth, but to interpret the ways through which football talent is constructed and reproduced by the Italian sporting press. These ontological and epistemological assumptions informed the inductive qualitative inquiry used in this study, which we outline in a dedicated section of the manuscript.

Moreover, in line with our interpretivist orientation, it is important to acknowledge our positionality. Our research group is currently investigating the talent development ecosystem in Italian football, including topics such as the presence of birth advantages and explorations of how the characteristics of local communities can influence players' developmental experiences. For this reason, as researchers interested in and embedded within the particular cultural environment of Italian football, our positionality has encouraged reflexivity throughout the research process and has played a key role in shaping both translations and what emerged as salient from the analyses carried out. It has also supported us in contextualizing findings within the broader ecosystem, thereby facilitating a nuanced sociocultural understanding of the talent narratives.

Furthermore, our philosophical orientation informed the theoretical perspective we used for this article, founded on an ecological approach (which is explained below), whereby we did not consider football talent as an object fixed through time that can be objectively measured (Baker et al., 2018), but as a socialization process (Araújo et al., 2023), produced and reinforced by interactions happening with human and non-human actors, historically changing, open-ended, and contextually embedded (Morganti et al., 2024c).

### 2.2 Theoretical considerations: expertise development and intentionality in sporting contexts

The theory of ecological psychology (Gibson, 1979) presents an innovative perspective able to overcome the traditional dichotomies (e.g., mind and body; perception and action; Fajen et al., 2008) and has been used recently by authors to reframe the development of sporting expertise (Araújo et al., 2023). Specifically, ecological psychology can help comprehend how learning and skill acquisition processes are socially constructed through multiple interactions between the performer and the environment, therefore, offering the opportunity to investigate and comprehend sporting and non-sporting influences on the development of sporting expertise. Recent research on the area has underlined how political, sociocultural, historical, and economic factors play an important role in shaping regular ways of doing and being (e.g., developmental practices and behaviors), thus defining expectations, learning, and performance outcomes (Ejekwumadu, 2023; Morganti et al., 2025b; O'Sullivan et al., 2023).

Based on ecological psychology, we perceive the world in terms of what opportunities for action it offers us, through the theory of affordances (Gibson, 1979). Affordances have material values, meaning they are objectively present in the environment; however, they are defined and emerge at the level of the organism-environment relationship, whereby we can perceive and act on them based on our body measures, action capabilities, or task constraints (Araújo et al., 2019; Gibson, 1979). Indeed, authors distinguish between the term landscape of affordances and the term relevant field of affordances (Kiverstein and Rietveld, 2021; Kiverstein et al., 2021). The former refers to the totality of affordances offered by the environment, whereas the latter refers to the possibilities for action that stand out or can be perceived as salient or significant compared to the others (Kiverstein and Rietveld, 2021). Further elaborating on the theory of affordances, and of particular concern for our study, the Skilled Intentionality Framework (SIF; van Dijk and Rietveld, 2016) argues about the social values of affordances, which are embedded in a particular form of life, whereby their relevance or significance is dependent on socio-cultural and historical factors (e.g., sociomaterial entanglement; Vaughan et al., 2021).

According to the SIF, specific cultures of reference, characterized by sets of promoted values, beliefs, traditions, or customs, can indirectly shape the landscape of affordances, highlighting certain opportunities for action over others, thus defining the relevant field of affordances (O'Sullivan et al., 2022, 2023; Vaughan et al., 2022). In this way, athletes' perceptual attunement is directed toward using opportunities for action that resonate with cultural significance, suggesting how intentionality emerges due to and is related to the whole athlete-environment relationship (O'Sullivan et al., 2021; Rossing and Skrubbeltrang, 2017). Research has proposed that this interaction forms the basis for comprehending skill acquisition processes, preferences, expectations, as well as individual and collective playing styles from a contextual perspective (Morganti et al., 2025b), ultimately leading to the development and reproduction of specific practices, behaviors, attributes, and characteristics (Uehara et al., 2021).

In line with this, researchers have used the (Bronfenbrenner 1995) bioecological model of human development to inform their methodological framework and organize their knowledge, helping to identify relevant constraints on the development of athletes (Uehara et al., 2016). Specifically, Bronfenbrenner proposed the process-person-context-time interrelationship to serve as the basis for investigating human development (Bronfenbrenner and Morris, 2006). Based on the bioecological model, human development results from the interactions of active human beings (i.e., person) with their immediate settings, including human and non-human actors (i.e., proximal processes), which are influenced by the broader societal contexts in which these settings are embedded (Bronfenbrenner and Morris, 2006). Going more in-depth, the environment is conceived as a four-level nested system (i.e., microsystem, mesosystem, exosystem, and macrosystem). The microsystem is the innermost level, including activities and experiences in which the individual is directly engaged (e.g., school, family, or sports clubs). The mesosystem refers to the relations between two or more microsystems (e.g., parental involvement in sports or in school). The exosystem consists of settings in which individuals do not directly and actively participate, but which can still indirectly influence their development (e.g., the social network of the family or neighborhood characteristics). Lastly, the macrosystem is the outermost level of the model, which encompasses the socioeconomic, political, and historical patterns existing at the cultural level. Relevant to this study, past research has proposed that newspapers can act as distal and exosystemic influences on human development (Henriksen and Stambulova, 2017; Morganti et al., 2025b; O'Sullivan et al., 2023), able to reinforce hegemonic beliefs about talent (i.e., what and who counts as talent and what and who does not; Monsees, 2024).

### 2.3 Materials and analysis

We collected articles in Italian using a Google News search with the keywords “calcio” [football] and “talento” [talent]. We started data collection in October 2024. Therefore, to gain a coherent contemporary understanding of the football talent narratives in the Italian sporting press, we considered only articles written between October 2023 and October 2024. Assuming national sporting outlets would have a greater social resonance and cultural impact than general and local ones, we considered only articles from La Gazzetta dello Sport, the most read and downloaded Italian sporting newspaper, with a daily 1.77 million readers, and an additional 409,000 readers accessing the digital edition (La Gazzetta dello Sport, 2025). The search resulted in 39 articles. After scanning each article twice, we made a purposeful selection of 26 “information-rich” articles, that is “those from which one can learn a great deal about issues of central importance to the purpose of the study” (p. 230; Patton, 2002). In other words, in the analysis, we included articles containing in-depth information regarding youth players' individual histories and/or developmental experiences, excluding articles that focused on the team-level only or did not meet those criteria.

It is worth noting here that since we focused our analysis on a single newspaper, several articles were written by recurrent journalists specializing in youth football-related topics, who were appointed or invited by La Gazzetta dello Sport to write about talent in football. In total, 16 different journalists have written the 26 articles that were included in the analysis. This may result in limitations, as some journalists have authored more than two articles, and therefore, their perceptions may be more represented than others. However, considering our aim was not to capture inter-journalists differences in football talent narrations in Italy, but rather to interpret the exosystemic role played by the press as a whole, what is relevant for us is that the totality of the articles included in the analyses expresses the construction of football talent narratives disseminated by La Gazzetta dello Sport, which is the Italian sporting newspaper with the highest readership, thus the one that can facilitate cultural reproduction more than any other newspaper.

We analyzed the articles in their original language, Italian, which is the first language of the lead author. Then, for reporting purposes, we carefully translated relevant quotes from the articles into English. We considered the option of anonymizing the names of players and authors of the newspapers' articles. However, it is worth noting that all the information presented in this study is freely available in the public domain (Accessed legally through subscription) and cited accordingly. In addition, we collected no personal or sensitive data. Therefore, no approval by an ethical committee was required.

We analyzed articles through inductive content analysis, meaning that analysis was “built up inductively, from a close reading of the texts rather than searching the text for a pre-determined list of content items” (Vears and Gillam, 2022, p. 112). First, we read and re-read each article to get a whole and general understanding of what they were talking about (Morse, 1994). In this early step, we wrote down initial impressions, including: what is the text talking about? What stands out? What message did the text leave you with? (Erlingsson and Brysiewicz, 2017). Second, we divided the articles into meaning units, asking ourselves, “What is this section about?” (Erlingsson and Brysiewicz, 2017; Vears and Gillam, 2022). Third, the first round of coding was undertaken, a preliminary piece of analysis which aimed to develop codes acting as descriptive labels for meaning units, grouping them into categories (Erlingsson and Brysiewicz, 2017; Vears and Gillam, 2022). Fourth, we conducted the second round of coding, which involved a closer look at each meaning unit to develop sub-categories through fine-grading coding (Vears and Gillam, 2022). It is worth noting that in inductive content analysis, the process of coding is iterative, meaning that new aspects will continue to emerge from the data as more documents will be analyzed (Vears and Gillam, 2022). For this reason, both the first and second round of coding included adjustments, refinements, and re-codifications as new information emerged from the dataset (Erlingsson and Brysiewicz, 2017). Finally, we interpreted data by “synthesizing and connecting the categories to create an overall narrative for the reader” (Vears and Gillam, 2022, p. 122).

## 3 Findings and discussion

In analyzing press narratives through the lens of ecological psychology and the SIF, we attend to how these texts act as a distal and non-sporting constraint that indirectly constructs a relevant field of affordances for aspiring football players and their families. Specifically, we present what developmental pathways, interpersonal relationships, performance standards, progression curves, and attributes are perceived and portrayed as salient opportunities for action, or in other words, as routes to being recognized as talented. We can argue how these narratives do not merely describe talent but prescribe specific ways of becoming talented, thereby orienting the intentionality of athletes, parents, and coaches who seek to navigate the Italian football ecosystem. As shown in Table 1, our inductive content analysis revealed four overarching categories, each containing two to three sub-categories, revealing distinct patterns in how the Italian sporting press constructs their prescriptive narratives, which we discuss in turn below.

**Table 1:** Categories and sub-categories emerged from the inductive content analysis displaying football talent narratives in the Italian sporting press.

Categories	Sub-categories
The early steps of football talent	Early start and father as mentor and gateway to early participation
	Early selection and specialization in football
Developmental pathways and career trajectories of football talent	Early achievements and performance progression
	Playing up (competing in older age categories)
	Early exposure to senior-level football
The attributes of football talent	Body proportions and physical attributes
	Technical and tactical skills
Psychological characteristics
Socially mediated validation of football talent	Public recognition and market value
	Youth international selection

### 3.1 The early steps of football talent

The narratives associated with the category *The Early Steps of Football Talent* emphasized the initial contacts with the sport and included the following sub-categories: (a) Early Start and Father as Mentor and Gateway to Early Participation and (b) Early Selection and Specialization in Football.

#### 3.1.1 Early start and father as mentor and gateway to early participation

The sub-category *Early Start and Father as Mentor and Gateway to Early Participation* introduced the concept of precocity, defined here as participation and involvement in football activities from a very young age: “he started training [and playing] for the team of his little town very early [four years old]” (Maresca, 2024a). Notably, articles not only recounted the early start but also underlined the quality of the developmental environments, as shown in this example: “He was five when he started playing organized football with one of the most prestigious football teams in Rome” (Pugliese, 2024).

The analysis revealed that articles gave particular attention to the role of the father, often framed as a mentor able to initiate his son into the game of football. Indeed, the father was narrated as a central and active figure, who as a past amateur or professional player instilled in his son the lure of football: “His love for football, inherited from his father, showed early when he was just eight, and started playing with the local team” (Seu, 2024a). This included being both a mentor and a source of inspiration:

[He] scores to make it 2–1 against Savona, then runs toward his son Tommaso, who is cheering behind the fence. He's eight years old, blond like his dad, and smiling like someone who's just seen his superhero win yet another battle. […] Ten years later, the roles have reversed: Tommaso is the one having fun and scoring goals, while his father Raffaele watches proudly from the stands. (Maresca, 2024c)

Credit goes to his father, a former amateur goalkeeper, who decided to have him treated by a specialist [the player suffered from backpain]. Simon used to play in defense, but he was dropped, partly because of the pain, partly because he wasn't seen as a good fit, and so he decided to try goalkeeping. Out of a sense of emulation. (Cascini, 2024)

In line with this, fathers played an important role in pushing their sons to start their development journey from a very young age, as analyzed here:

For Zanos, football is a family affair. His father, Petros, played for many years as a goalkeeper, and once he hung up his gloves, he devoted himself entirely to his son's development. Training sessions in the yard, specific drills on the pitch, physiotherapy, and strength work. He did everything to secure a future in football for his child. (Maresca, 2024b)

These findings align with research in this area, which highlighted that fathers involved in youth sports are often active figures who engage with coaches, provide strategic advice to their sons, assess the quality of training and tactics, or criticize performances or commitment (Coakley, 2006). In English youth football, Magee (2018) identified and described different father types, distinguishing between fathers who mainly supported their sons through transports and time commitments, and active fathers who closely followed training and match play, discussed coaches' actions, offered tactical advice, and displayed higher expectations for their son's potential. In this regard, the fathers narrated by the press can be considered active and expert figures, supporting football involvement in both informal and formal environments. These fathers often act as coaches, inspirational figures, or even as agents, who “patiently put together and sent out videos, as well as [arrange] the trials” (Seu, 2024b) for their sons. Consistent with these observations, Erikstad et al. (2025), investigating the father-son relationship within Norwegian professional football, found that past professional fathers were perceived to play a major role in the development of their sons in many aspects, including shaping their aspirations to succeed, providing opportunities, and social visibility, or acting as a competent companion or private coach able to support their participation.

Overall, the Italian sporting press helped characterize the salient features of Italian football talent's families, described as sporting, supportive, and collaborative environments: “Last year, he was traveling almost 4 h round-trip for training sessions and matches. His mother, Eleonora, took him everywhere—including 2 years ago, during his spell with Cremonese” (Maresca, 2024d).

The four Manzoni children are all footballers: Matteo, the eldest, has moved to the United States to study and plays for his college team. Francesco, the third-born, is 15 and currently with Giana Erminio. The youngest, four-year-old Leonardo, has just started football school. Alberto, too, began playing at a very early age. (Maresca, 2024a)

As a critical implication, the results of our qualitative analysis indicated the reproduction of traditional gender ideology related to parental roles, through youth sports. Consistent with previous research, which has highlighted that mothers in youth sports are mainly institutionalized as launderers, chauffeurs, cooks, and sources of emotional support (Coakley, 2006), the Italian sporting press's narratives render them invisible. They are almost absent from articles and appear only once, where a mother is cited as a driver. Similarly, in Ghanaian youth football, Acheampong et al. (2024) investigated women managers' perceptions of their roles, revealing that they operate within a male-dominated environment that limits participation opportunities for youth female footballers and undermines their credibility as managers within football.

More broadly, our findings also indicate that the Italian sporting press largely excludes women's football from talent narratives, which are reported exclusively from a male perspective. This gender disparity in sports press coverage has been reported in prior research (Simões Mello and Hilgemberg, 2025; Monsees, 2024) and can negatively affect the visibility of women players, limit the rewards for their accomplishments, and reinforce the positioning of men's football as the normative model against which women's football is evaluated (Gang et al., 2025).

#### 3.1.2 Early selection and specialization in football

Subsequently, the press contributed to a first transformation of the concept of precocity, which, within the sub-category *Early Selection and Specialization in Football*, progressed from childhood involvement to the ability to rapidly reach a higher level of performance standard. Indeed, the press remarked on the early selection and specialization of the players, who, thanks to their initial start with their local football team, benefited from competitive advantages, whereby they outperformed their peers and were scouted by a bigger and more prestigious one: “[he] joined the Rossoneri [AC Milan] in 2014, at the age of seven. [Before] he played as a kid for two local football clubs, showing his early talent and catching the eye of scouts” (Antonelli, 2024a). Importantly, the press highlighted how the new team was more selective: “he joined the LUV Graz youth academy [at the age of eight], and 2 years later moved to the much more selective one at Sturm” (Seu 2024a), and offered professional developmental environments: “… [he] stayed there [its local football club] until the age of nine, when he was picked up by AS Roma and brought straight to Trigoria [the club's professional training center]” (Pugliese, 2024).

We suggest interpreting these findings within the wider context of youth sporting systems, which generally prioritize higher performance standards and promote practices of early identification and specialization (Christensen and Sørensen, 2009). Indeed, the enduring myth that expertise requires 10,000 h of practice (Ericsson et al., 1993) means children are often encouraged to start their football-specific training from a very young age to accumulate hours of practice and increase their chances of being considered talented. In this context, early access to organized activities may be linked to developmental advantages on different time scales, including increased interactions with peers and coaches, and faster football-specific skill development (Kelly et al., 2022a). These factors can contribute to early performance differences and competitive success, which may, in turn, be associated with increased motivational level, social visibility (Söderström, 2025), and higher chances of achieving senior success (Baker and Wattie, 2018). Overall, this body of knowledge reveals that the achievement of football expertise cannot be solely derived from training age and/or accumulated hours of practice and competition (Ford et al., 2009; Haugaasen and Jordet, 2012; Hendry and Hodges, 2018), but rather by entrance into high-performance, high-quality, and challenging learning environments (Bjørndal et al., 2018; Sweeney et al., 2025).

Through the lens of ecological psychology, we can argue that the Italian sporting press acts as a distal and exosystemic influence, reiterating culturally ingrained behaviors. Here, the media reflect traditional gender roles and gender exploitation, indirectly influencing parenting models and what is perceived as good fatherhood and motherhood. For instance, in these press narratives, mothers perform the invisible reproduction of capital (Federici, 2020; Lonzi, 2023), which, in this case, involves the reproduction of youth sporting practices (Gözmen Elmas et al., 2025), whereas fathers are active agents who, through their choices and actions, directly impact their son's development and career progression (Erikstad et al., 2025; Magee, 2018). Moreover, the Italian sporting press echoes the structural organizations and underlying logics of youth football systems, in which entrance and specialization in higher-quality developmental environments play a major role. This way, childhood engagement is retrospectively interpreted as a competitive advantage and predictor of future success.

Accordingly, from the perspective of the SIF, the press's narratives, reinforcing parental roles, and normalizing early start, selection, and specialization practices, make a significant contribution to shaping the relevant field of affordances for parenthood and aspiring professional players, potentially reiterating gender exploitation and the proliferation of developmental advantages in the Italian football context.

### 3.2 Developmental pathways and career trajectories of football talent

The narratives associated with the category *Developmental Pathways and Career Trajectories of Football Talent* involved the passage from early engagement to the first stages of development and included the following sub-categories: (a) Early Achievements and Performance Progression, (b) Playing Up (Competing in Older Age Categories), and (c) Early Exposure to Senior-Level Football.

#### 3.2.1 Early achievements and performance progression

The sub-category *Early Achievements and Performance Progression* emphasized performance progression and immediate career achievements: “In the Under-20 World Cup with Italy, Cesare played a key role, finishing as a runner-up and claiming both the Golden Ball (Best Player) and the Top Goalscorer award” (Vocalelli, 2024). In these narratives, the press underlined how football talents, once recruited by a higher-level club, continued to outperform peers regularly and collected subsequent selections, as illustrated in the excerpt below:

[He] joined Roma in 2013, working his way through every level of the youth academy and winning the Under-15 championship in 2019 with a stunning goal in the final against Milan. He came close to another title with the Under-16s the following year [...] and lifted the trophy again in 2021 with the Under-17s. (Zotti, 2024)

Interestingly, youth players' ability to stay in the system is often narrated as a potential sign of predestination, or innate propensity toward football and the achievements of football expertise:

[He] has grown up with Atalanta in his blood: he's been wearing the Nerazzurri shirt since he was nine years old [when he was scouted]. At the stadium, he used to watch his idols from behind the goal, [he was] one of those ball boys dreaming of making his Serie A debut one day. (Maresca, 2024a)

His world has always had red and black in the background. [He] grew up in Milan, and his bond with the Rossoneri grows stronger day by day. […] From his first matches when he had eight, to the dream of San Siro. (Antonelli, 2024b)

Moreover, articles, when recounting the developmental pathway of the talented football players, highlighted how their progression through the system, as well as the recognition of their talent by public opinion, are linked to obtaining early career achievements, performing at given standards when it matters, and accumulating outstanding match statistics:

[He had] the joy of winning the Youth Ballon d'Or in 2019 (...) in the Under-13 category. (...) [He] kept growing, eventually winning the Under-16 title last year, and proving decisive in the final against Fiorentina, which ended 3–2, with him scoring Roma's opening goal. (…) His current campaign with the Under-17s (…) where he has so far scored 15 goals (3 in the playoffs) and provided 6 assists in 23 matches. (Pugliese, 2024)

Last year he was with the Under-17s and impressed everyone, scoring as many as 30 goals. He finished as the league's top scorer, though his team fell in the Scudetto semi-final against Inter. (…) He keeps scoring goals for fun in the youth teams (…) four in his last three matches, and he has no intention of stopping. (Maresca, 2024c)

It is essential to situate these findings within the broader literature on talent identification and development. Research has already presented how youth football practitioners tend to infer their opinion of players' potential or talent by analyzing their current performance values (Gilson et al., 2025). For instance, in English youth football, Barraclough et al. (2024) highlighted how coaches' perceptions of potential are linked to match performance and athleticism. Nevertheless, this performance-centered approach to talent identification and development has been shown to disregard the individual, non-linear, and dynamic nature of development (Liu et al., 2006) and has been linked to the potential proliferation of selection biases (Baker et al., 2018; Till and Baker, 2020). In more detail, a vast body of knowledge produced in football highlighted how performance outcomes at the youth level are associated with developmental advantages, including relative age effects (Morganti et al., 2025a; Brustio et al., 2026), birthplace effects (Hernández-Simal et al., 2024), and biological maturation (Hill et al., 2020).

Importantly, recent findings have revealed that the Italian football context is prone to such biases. For instance, Morganti et al. (2024b,a) revealed that youth players born at the end of the selection year (i.e., from October to December) and players born in Southern Italy have a decreased likelihood of reaching the national youth and senior squads, as well as competing in the Under-17 national championship, compared to their counterparts born at the beginning of the selection year and/or in Northern and Central Italy. Consistent with theoretical accounts explaining the underlying mechanisms of relative age effects and birthplace effects (Côté et al., 2006; de Souza et al., 2025; Kelly et al., 2022a), the authors suggested that an overemphasis on early results and performance achievements, as well as the interactions between historical, political, and cultural factors characterizing Italy, can be linked to the current inequalities of developmental opportunities and outcomes found in the Italian football context. For this reason, we suggest that by promoting early performance and competitive achievements in youth football, the press's narratives not only describe developmental pathways but legitimize performance-centered criteria as indicators of talent, without considering the inherent fallacies of such a model of talent selection, thus potentially contributing to the reiteration of selection biases and reinforcing the current inequalities.

Moreover, the press's narratives tend to frame youth players' entrance and progression through the ranks as a sign of their imminent professional success. This approach does not take into consideration current research in the area, which has consistently highlighted low rates of conversion from youth to senior football (Verbeek et al., 2026). For instance, in Italian football, Morganti et al. (2024a) investigated the future career status of Italian-born players competing in the Under-17 national championship 5 years after their youth season and reported 82% of them continued playing at the recreational level or dropped out of football entirely. Nevertheless, articles do not include dropouts or less successful narratives, while preferring to promote an almost linear relationship between early and senior success.

#### 3.2.2 Playing up (competing in older age categories)

Within the sub-category *Playing Up (Competing in Older Age Categories)*, the analysis also revealed a second transformation of the concept of precocity, which continued to evolve through the narratives, and was now related to the idea of rising quickly through the age groups. More specifically, the press underlined that talented youth football players, due to their higher-level performance outcomes, are often called up to permanently “play up” an age-group, training with and competing against older peers, as encapsulated here “Young, talented, full of potential, and a specialist at fast-tracking his development” (Seu, 2024c).

Last season he was a regular starter for the Under-16 side, while also making several appearances with the Under-17s, playing above his age group. The same happened this year, as he played with the Under-19 team, which came just short of a historic Youth League triumph (losing only in the final to Olympiacos) and reached the Scudetto playoffs. (…) 25 appearances with the Under-19 side, across the league, Youth League, and Coppa Italia, recording 7 goals and 2 assists. (Antonelli, 2024a)

In youth sports, when youth athletes show they are more skilled than their same-aged peers, they may be allowed to train and compete in higher age groups, experiencing more challenging learning environments (Kelly et al., 2021; Goldman et al., 2021). Goldman et al. (2022) explored the perception of Canadian youth football players regarding playing up and revealed that it involves a combination of challenges and progress. Specifically, playing up was considered by players as a milestone in their developmental pathway, which could motivate them to develop their football expertise. However, the perception of playing up varied greatly from player to player (Goldman et al., 2022). Indeed, several drawbacks are associated with this practice, including social isolation, feelings of being overwhelmed, discouragement, or increased risk of injury perception (see Kelly et al., 2023 for a discussion on this), which could potentially lead to burnout or dropout (Gustafsson et al., 2018). In this regard, results from our qualitative analysis revealed that the Italian sporting press promotes playing up as a performance and potential indicator rather than as a strategy to encourage individual and tailored development (Kelly et al., 2021).

#### 3.2.3 Early exposure to senior-level football

Another important factor in being recognized as talented by public opinion emerged within the sub-category *Early Exposure to Senior-Level Football* and was linked to players' ability to accumulate early exposure to senior football, whereby narratives often underlined call-ups to the senior teams received by youth players, in the form of training or match experiences, as a result of their higher performance standards: “This summer, it was already clear that [he] had fast-tracked every stage of his development. He joined the first-team pre-season camp and even scored in a friendly against Feralpisalò” (Maresca, 2024b).

That's why he was promoted to the Under-19s just after turning sixteen. His impact there was stunning as well: 10 goals in his first 6 appearances, followed by the inevitable call-up to the first team, where he also netted his first official goals in Ligue 2. (Seu, 2024c)

Indeed, the press recognized the difference between youth and senior football, as illustrated here: “[On the first team there is] a faster pace, greater attention to detail. The first team is work. A battle. No jokes, no loss of focus: if I play, you don't. It's pure competition” (Di Feo, 2024), and therefore, highlighted how training and competing against senior players can support football talent development:

[He] knows that the road to his dreams is still long and winding, and that the second team [playing in the professional third division] is the ideal place to gain experience and give his career an extra push at this stage [he is eighteen]. (Albanese, 2024)

Research revealed that the youth-to-senior transition requires youth players to adapt to higher match and training intensity, different playing styles, greater physicality, and augmented pressure on winning matches (Lundqvist et al., 2022; Swainston et al., 2020). In line with this, English coaches highlighted that youth players training in a first-team environment need to cope with technical and tactical requirements and speed of play, as well as adhere to certain regular ways of doing and being, such as displaying enthusiasm while playing and presenting themselves well in terms of their training kit and boots to be perceived as professionals by senior players and team management (Røynesdal et al., 2018). For this reason, practitioners consider having the academy and the first-team training on the same site a resource that could facilitate the youth-to-senior transition (Lundqvist et al., 2022). Indeed, through increased and earlier contacts with the first-team environment, youth players can potentially speed up their acculturation process, developing the required psychosocial attributes to transition to the senior level. Accordingly, results from our qualitative analysis, which indicated that the Italian sporting press considers exposure to senior football during the formative years both evidence of potential and as a mechanism enabling its fulfillment, should be interpreted within this wider context.

Adopting an ecological stance, we propose that the Italian sporting press represents an environmental constraint, reflecting broader culturally derived norms, embedded in deterministic assumptions, which promote an almost linear and predictable relationship between early ability levels, current performance standards, developmental progression, and future achievements (Morganti et al., 2024c). In more detail, these narratives contribute to the institutionalization of a performance-centered approach to talent identification and development by foregrounding precocious success. This way, the press reproduces a concept of football talent defined by constant ranking. In this framework, potential is related to adherence to pre-defined developmental pathways and the consistent outperformance of peers.

Accordingly, through the SIF, we suggest that the press's narratives indirectly guide the intentionality of the system (i.e., coaches, practitioners, parents, or players) directed toward early performance attainments and success, indicating relevant opportunities for action for youth players developing to the professional level.

### 3.3 The attributes of football talent

The narratives associated with the category *The Attributes of Football Talent* highlighted the individual characteristics of the players, which sustained their performance outcomes and are narrated as key features of their (innate) potential, including the following sub-categories: (a) Body Proportions and Physical Attributes, (b) Technical and Tactical Skills, and (c) Psychological Characteristics.

#### 3.3.1 Body proportions and physical attributes

Within the sub-category *Body Proportion and Physical Attributes*, the press often remarked on displaying ideal body proportions and expressing physical attributes as essential for outperforming peers and being prepared for the senior level, indicating these are important factors to consider when evaluating players:

Some talents, no matter how special, are considered too small for top-level football and are therefore labeled as having little potential. So, instead of giving them a chance, you end up looking elsewhere, maybe signing players who are less gifted but taller, stronger, and more physically imposing. (Di Feo, 2023)

You have to use your strength, hold the ball up, act as a focal point, get into the box and battle it out with thirty-year-old defenders standing six feet tall (…) Thirty years ago, if you didn't have great physical attributes but had good technique, you could still play football. Not anymore (…) Even the most technically gifted players need solid athletic qualities. (Di Feo, 2024)

These characteristics are narrated as able to facilitate selection and progression through the system: “[His] explosiveness and physical power, despite facing boys all older than him [he was playing up the age groups] left the entire Amiens coaching staff stunned” (Seu, 2024c).

It was clear from his childhood that he was destined to be a top striker: “Playing five-a-side or seven-a-side, someone with [his] physique makes all the difference. In his early years with Castiglione [a local youth team], he'd run with the ball at his feet and head straight for goal (…) he makes his physique count and has good acceleration. With his height [he is sixteen and measures six feet tall], he's hard to stop when he goes up for a header.” (Maresca, 2024d)

Consistent with these findings, in Dutch youth football, Platvoet et al. (2020) showed that from the very initial stages of development (i.e., Under-11) professional academies tend to prefer players with higher physical and technical abilities. Within the same context, Fortin-Guichard et al. (2022) found that selected players were bigger (i.e., taller and heavier) and had better physical fitness compared to their non-selected peers. Nevertheless, research has already highlighted the potential detrimental effects of this selection bias, which could disadvantage the development of late-maturing players (Gilson et al., 2025). For instance, in Italian youth football, Toselli et al. (2022) indicated that earlier maturing players were taller, heavier, and scored higher on physical performance testing. Indeed, as noted by Hill et al. (2020), advanced maturity status can significantly predict higher grades during matches in English youth football. This way, sporting systems can potentially cause the reproduction of false-positive errors (i.e., the progression through the system of high-performance and low-potential individuals) and the premature exclusion from the pathway of low-performance and high-potential individuals (i.e., false-negative errors; Baker et al., 2018; Gilson et al., 2025).

#### 3.3.2 Technical and tactical skills

The press also highlighted the need for showing higher technical abilities, which were defined within the sub-category *Technical and Tactical Skills* as the ability to dribble pass opponents, or move and pass the ball well, and tactical skills, intended as game vision, to be recognized as talented by public opinion: “[He is] a creative box-to-box midfielder with refined technique and natural flair (...) dribbling is one of his trademark skills” (Pugliese, 2024); “He's a left-footed playmaker, known for his flair and technical quality” (Pietrella, 2024).

Another individual playing characteristic highly valued by the Italian sporting press is the versatility of the youth players, that is, their ability to play in different field positions: “Versatility is one of [his] best qualities: he can play as a winger, a right midfielder, or be moved into a central role. He's not afraid to adapt” (Maresca, 2024b).

He does so by adapting to multiple roles and positions, proving to be a valuable utility player both in a 4-3-3 and in a 3-5-2 system. His natural habitat is as a right midfielder or wing-back in a five-man midfield (...), but [he] can also comfortably operate as a wide forward on either flank or, alternatively, on the left wing. (Seu, 2024d)

This way, players can functionally respond to different coaching strategies, tactics, and specific requests, possibly maximizing their developmental opportunities and playing minutes.

Importantly, when narrating and analyzing youth players' physical, technical, and tactical attributes, articles tended to compare their individual characteristics and style of play to current or past professional players, almost as if talent is a concept already encapsulated in someone else and a model to be reached:

His imposing physique recalls that of Osimhen, with whom he shares his hometown of Lagos, but his technical and athletic traits are closer to Thuram's. [He] is more of a mobile, space-seeking striker than a pure target man, a forward who relies on speed and acceleration, combining them with excellent dribbling ability and a sharp eye for goal. (Seu, 2024c)

You could say that today, in terms of playing style, [he] has something of the very early Haaland about him. He's a pure center-forward: he loves to stay up front and touch the ball only when needed, striking decisively at just the right moment. (Pietrella, 2024)

In line with these findings, in Italian youth football, Abate Daga et al. (2024) recorded a positive correlation between players' playing time and the coaches' subjective ratings of their technical and tactical skills. Indeed, Kelly et al. (2020) underlined that match analysis statistics, such as dribbling completion percentage, average total touches, or pass completion percentage, and technical testing procedures, including slalom dribble, ball juggling abilities, or shooting accuracy, discriminated between coach perceived higher and lower potential players in English youth football. Moreover, research in the area has already emphasized how selection decisions and perceptions of potential are highly influenced by practitioners' preferences and past experiences (Johnston et al., 2025). For instance, in Swedish football, Lund and Söderström (2017) highlighted that coaches guide their decision-making, looking for qualities that to a higher degree resemble those observed in players they have identified in the past who eventually developed into senior professionals.

#### 3.3.3 Psychological characteristics

As revealed by the sub-category *Psychological Characteristics*, the press also placed particular attention to the psychological dimension of youth players, including in their football talent narratives their ability to cope with pressure, ambition, and dedication. These characteristics are emphasized as they are considered essential factors that support players in reaching their potential. For instance, the press highlighted youth players' coping strategies, recognizing their importance in guaranteeing they can perform when it matters the most, thus facilitating their progression through the talent pathway:

[He] also stands out for his strong personality and is certainly not one to crumble under pressure (…) He's not the type to shy away from a battle either, [he can be considered] a player with remarkable composure. (Seu, 2024d).

In a similar vein, the ambition and dedication of youth players, who exhibit athletic identity and willingness to train, compete, and follow instructions, are often narrated as evidence of their desire to achieve the highest level of performance, which could increase their likelihood of attaining the professional level. Indeed, the press emphasized when youth players showed a higher level of commitment to their football career:

[He has always showed] drive and determination (…) [He is young and has] got plenty of time ahead of him, but he knows that nothing will come by chance, it will all depend on the quality of his work. (Albanese, 2024)

[His past coach recalled] “What's most impressive about [him], is his ability to improve with every game. He keeps taking steps forward and plays with an intensity that's truly uncommon. I've seen very few youngsters in my life as talented, and as driven, as he is.” (Seu, 2024c)

Another important factor often considered by the press when writing their football talent narratives is the youth players' ability to balance academic and football duties. Specifically, the balance between the two was particularly used by the press when they aimed to highlight the psychological maturation of the youth players, possibly indicating their responsibility toward obligation and their conformity to norms or rules:

[He] wakes up every morning at 7 to attend classes, trains in the afternoon, and study in the evening. He speaks four languages, Italian, English, French, and Spanish, and never skips a day at school. Last year, [he] also received the Brembo Award, given by the club to youth players who stand out for their behavior off the pitch and their academic performance (Maresca, 2024a).

In English youth football, (Kelly et al. 2024) highlighted how coaches' perception of players' potential was linked to their ability to cope with performance and developmental pressures. In another study within the same context, these factors were also the only key differentiator between those Under-18 players who received their first professional contract and those who did not (Kelly et al., 2022b). In line with this, in German youth football, Wachsmuth et al. (2024) underlined that among Under-17 players, those who developed as professionals 4 years after their youth season recorded significantly higher values for hope for success, competitiveness, and self-efficacy, as well as lower scores for fear of failure, anxiety, and disruption of concentration. Indeed, in football, the progression to professional status may require players to develop coping strategies to overcome obstacles, as well as learn to adjust after setbacks or transitions (Collins and MacNamara, 2012).

Furthermore, research has reported that youth players in high-performing learning environments are often required to exhibit a single-minded dedication to their football development (Champ et al., 2020; Christensen and Sørensen, 2009). In this regard, Clarke et al. (2018) revealed how players acknowledge the need to display the right attitude, passion for the game, and willingness to train to be retained by the academy system.

Therefore, from an ecological perspective, we suggest that the Italian sporting press promotes key individual characteristics of football talent in Italy that are related to broader ideals and beliefs, including the idea that physical attributes, specific set of skills, or psychological characteristics can differentiate between players with the potential to succeed at the professional level and those who do not. This way, the Italian sporting press acts as a distal influence on the development of Italian football players, contributing to shaping what talent should look like. For this reason, through the SIF, we can argue that by defining the salient features and behaviors of football talent, including working ethic, responsibility, and specific physical and technical attributes, the press's narratives can indirectly define standards that aspiring youth players should align with, as well as direct coaches' and practitioners' preferences and expectations and guide their selection decisions and training procedures.

### 3.4 Socially mediated validation of football talent

The narratives associated with the category *Socially Mediated Validation of Football Talent* underlined the social dimension of talent in football and included the following sub-categories: (a) Public Recognition and Market Value and (b) Youth International Selection.

#### 3.4.1 Public recognition and market value

Within the sub-category *Public Recognition and Market Value*, the press emphasized when players received public recognition of their talent from important others, who, considering their younger age, publicly praised youth players for their abilities: “[The professional coach] was in love with him: “Carboni is a super talent,” he [the professional coach] once said. [He also added:] “He needs to be protected. (…) He has a great future; let's enjoy him” (Pietrella, 2024).

[When asked about a young AC Milan player who had particularly impressed him], the former Rossoneri defender said in an interview with La Gazzetta [dello Sport] last year, well ahead of anyone else: “A youngster who impressed me? [Him], born in 2007, from Milan. He's young, but he reminds me of Foden. (Antonelli, 2024a)

Significant others can be represented by past professional players or expert and higher-level coaches, whose opinion, due to their experiential and practical knowledge, is highly valued and used as a formal proof of football players' talent by the Italian sporting press.

In line with this, another way youth players obtained public recognition as proof of their talent was by receiving interest from several higher-level professional clubs, which competed against each other on the football market to sign the players and were willing to invest large sums of money in their recruitment: “(…) when Inter let Cesare Casadei leave to join Chelsea. The economic choice was understandable, bringing in almost €20 million” (Vocalelli, 2024); or: “He had done very well with Olympiacos Nicosia, and many European clubs were tracking him. Even Sporting Lisbon and Braga tried to sign him, but the Granata club [Torino]was faster than the competition (Maresca, 2024b).

It's no surprise that scouts from across Europe began taking notice after the youngster's first appearances in Ligue 2. At Inter—to give some context to the latest transfer rumor—the idea last summer was to table an offer of around four million to bring him to Milan straight away. (Seu, 2024c)

As exemplified by Fürst (2024), talent is not confined to inherent abilities or possessed characteristics, but rather emerges through identification, recognition, and validation by others. These processes are essential to receive developmental support and have the highest likelihood of fulfilling one's potential. Within youth sporting systems, due to finite resources (Rongen et al., 2018), such as a limited number of professional and competent coaches or facilities, only a few (considered) talented individuals are selected and able to work on themselves in the sport-specific domain, receiving the best developmental opportunities and highest competition challenges. Hence, being identified as talented becomes a prerequisite for players to have the chance to develop their skills. In line with this, based on the results of our qualitative analysis, we suggest that the Italian sporting press constructs football talent through public recognition of one's ability by experts. This recognition is interpreted as a sign of possessing individual traits that distinguish certain youth players from their peers, simultaneously legitimizing economic investments. In this regard, in a football market characterized by increasing national and international competition related to scouting and talent identification procedures (Reeves and Roberts, 2019), the Italian sporting press contributes to the notion that receiving market interest from other European clubs maximizes public recognition and is positively associated with youth players' football talent.

#### 3.4.2 Youth international selection

The sub-category *Youth International Selection* remarked another significant sign of social validation that youth players can receive, which as reported in the Italian sporting press, was a call-up to their respective youth national team. Articles placed a lot of emphasis on players' ability to enter their national talent pathway, as this was the ultimate sign interpreted by the press that they are one of the best national players in their age group, thus deserving the label of football talent: “Last year, he was among the key players in winning the under-23 Africa Cup of Nations, and then debuted with Walid Regragui's [senior] national team in September” (Cordolcini, 2024).

His first time wearing the Italy shirt was on April 26, 2022, a 3-0 victory against Chile, with the Under-15 National Team. From there, he moved up first to the Under-16s and now to the Under-17 squad, where he shares the experience with three other Giallorossi [Roma] players: Cristian Cama, Alessandro Di Nunzio, and Federico Nardin. With them, he has become a key pillar of Massimiliano Favo's team, scoring a couple of goals before the Cyprus tournament and as many during the final phase of this European Championship. (Pugliese, 2024)

He entered the youth national team pathway at the age of 15. (…) He scored for the Cypriot Under-21 side in their last European qualification match against Austria. Last year, with the Under-19 team, he delivered an assist in the game against Romania after dribbling past half of the opposing team. (Maresca, 2024b)

Research has already shown the benefits of being recruited at the international youth level, which can provide early high-performing players with appropriate levels of challenge to support their career progression (Collins et al., 2016; Taylor and Collins, 2019). For instance, in Irish youth football, Sweeney et al. (2025) highlighted that players selected for a national developmental program felt exposed to higher-pressure environments compared to their grassroots clubs, including a greater degree of technical, tactical, and competitive challenges. Importantly, players perceived these demands as beneficial for their long-term development. However, the Italian sporting press tends to exclude from their narratives this conceptual idea, while preferring to use youth international selection as a marker of their social recognition, highlighting that players included in their narratives, who have been chosen by their respective national teams, are part of a (small) community of selected ones (Aarresola et al., 2017). In line with this, Söderström (2025) reported that, after being included in a national program, Swedish youth football players expressed feelings of augmented social status and self-esteem, which contributed to increased motivation for training. Accordingly, our qualitative analysis proposes that youth international selection emerges as a key factor contributing to greater social recognition by experts and visibility to scouts and top-level clubs.

From an ecological perspective, the Italian sporting press acts as a distal exosystemic influence, which increases youth players' social visibility and links their perceived potential to expert recognition and access to elite developmental and competitive opportunities. This way, the press's narratives not only resonate with experts' opinions, but also foreground specific developmental pathways or social relationships over others. In doing so, they may reinforce a binary distinction between “talented” and “non-talented” youth players on the fallacious basis of developmental advantages, despite research showing no clear relationship between youth international recruitment and later career outcomes (Verbeek et al., 2026).

Future studies should aim to compare data from different newspapers, including regional and local outlets alongside the national press, to explore potential intranational similarities or differences in talent narratives across contexts. Moreover, research could include exploring other sources of information, such as television programs, radio shows, specialized podcasts, or social networks, further examining how talent is considered, perceived, and narrated within the mass media.

## 4 Conclusions

In this study, we analyzed the construction of football talent narratives by the Italian sporting press. We interpreted the findings of our inductive content analysis through the lens of ecological psychology and the SIF. This way, we were able to investigate the sociocultural and historical influences that contribute to the reproduction of specific beliefs, habits, and practices related to talent identification, selection, and development processes in Italian football. Our findings revealed that the Italian sporting press plays a key role in providing meanings to the concept of football talent. Specifically, based on the results from our qualitative analysis, we suggest how football talent is constructed as emerging through adherence to an optimal and predefined developmental route. This includes receiving a given standard of social support from families during the formative years and from the system during the developmental stages, possessing qualities and characteristics that distinguish players from their peers, and obtaining validation and recognition from experts. In line with this, narratives unfold along a chronological timeline that follows players from childhood to youth and (eventual) senior levels, to explain and legitimize their football talent. Accordingly, from the perspective of ecological psychology and the SIF, we propose how the Italian sporting press functions as a distal exosystemic influence that contributes to shaping the relevant field of affordances within the Italian football ecosystem by prioritizing specific behaviors, customs, practices, and attributes as markers of “talent.” In doing so, the media highlights and prescribes particular ways of becoming talented, guiding the intentionality of parents, practitioners, and aspiring professional football players. Furthermore, by revealing dominant football talent narratives in the Italian sporting press, our study contributes to the ongoing discussion about refining conceptualizations of talent identification and development as complex and socially constructed processes. For this reason, we encourage sporting systems to reflect on the wider sociocultural contexts in which they are embedded when assessing and evaluating their current practices and outcomes.

## Data Availability

Publicly available datasets were analyzed in this study. This data can be found at: https://www.gazzetta.it/.
